# Factors Associated With Follow-Up Attendance After School Scoliosis Screening: A Pilot Cross-Sectional Study

**DOI:** 10.7759/cureus.110718

**Published:** 2026-06-12

**Authors:** Takuya Nagai, Syuji Kurogi, Kiyoshi Higa, Shota Amano, Takayuki Matsumoto, Takumi Takahashi, Naosuke Kamei

**Affiliations:** 1 Department of Orthopaedic Surgery, Faculty of Medicine, University of Miyazaki, Miyazaki, JPN

**Keywords:** adolescent idiopathic scoliosis, health information-seeking behavior, internet health information, parental awareness, school scoliosis screening, secondary screening

## Abstract

Background

Follow-up evaluation after school scoliosis screening is essential for early detection and timely management. However, not all children recommended for further evaluation attend secondary screening. This pilot study aimed to explore parental factors associated with follow-up attendance after school scoliosis screening.

Methodology

This pilot cross-sectional study was conducted as an anonymous online questionnaire survey, distributed via a Quick Response (QR) code to parents or guardians of 31,407 schoolchildren who underwent school musculoskeletal screening in Miyazaki City, Japan. Associations between parental awareness, information-seeking behavior, and intention to attend secondary screening were evaluated using Fisher’s exact test.

Results

A total of 3,072 parents responded (response rate = 10.0%). Among 217 children recommended for secondary screening, 146 (67.3%) had attended and 52 (24.0%) planned to attend secondary screening. Internet-based information-seeking behavior was significantly associated with intention to attend secondary screening (p = 0.006). Other factors, including general awareness of scoliosis, were not significantly associated with follow-up intention.

Conclusions

This pilot cross-sectional study showed that internet-based information-seeking behavior was associated with intention to attend secondary screening after school scoliosis screening. Although the low response rate and potential selection bias should be acknowledged, the findings highlight the potential role of accessible and reliable scoliosis-related information in supporting parental decision-making. Many parents expressed willingness to access QR-code-linked information; however, because this study assessed willingness rather than an intervention, the effect of such digital tools on actual follow-up attendance remains to be evaluated in prospective studies.

## Introduction

Adolescent idiopathic scoliosis is a common spinal deformity that typically develops during the growth period in children and adolescents and affects approximately 2-3% of the population [1,2]. Early detection is important because timely management, including observation or bracing, may prevent curve progression and reduce the need for surgical treatment [3-5]. For this reason, school-based screening programs for scoliosis have been implemented in many countries [6]. In Japan, school screening programs include musculoskeletal examinations aimed at identifying potential spinal deformities among schoolchildren. When abnormalities suggestive of scoliosis are identified during school screening, parents are usually advised to seek further evaluation at an orthopedic clinic or hospital [7].

Some studies have suggested that improving parental awareness of scoliosis may contribute to earlier recognition and detection of the condition [8,9]. In particular, scoliosis often progresses without pain and may not be easily recognized based on external appearance alone, which may lead parents to underestimate its clinical significance [5]. In addition, parents increasingly obtain health information through internet resources, and their information-seeking behavior may influence decisions regarding medical consultation [10,11]. However, it remains unclear whether parental awareness influences attendance at secondary screening following school screening. Therefore, the primary objective of this study was to investigate parental awareness of scoliosis and its association with intention to attend secondary screening after school screening. The secondary objective was to evaluate parental information-seeking behavior and parental willingness to access scoliosis-related information through online (Quick Response (QR)-code-linked) resources.

## Materials and methods

Study design and participants

This study was a pilot cross-sectional study conducted as an anonymous online questionnaire survey, in which parents or guardians accessed a survey created using Google Forms by scanning a QR code. The participants were parents of schoolchildren who underwent school musculoskeletal screening, including scoliosis screening, in Miyazaki City, Japan. The study protocol was approved by the Institutional Ethical Committee. The survey was conducted through the Miyazaki City Board of Education and distributed to parents of 31,407 schoolchildren from 72 elementary and junior high schools. Participation was voluntary and anonymous, and written informed consent was obtained electronically from all participants before completing the questionnaire.

Questionnaire

The questionnaire was designed to assess parental awareness of scoliosis, information-seeking behavior, and attendance at secondary screening following school screening. Parents were asked about the age and sex of their children, their awareness of scoliosis before school screening, and whether they understood that scoliosis cannot always be reliably identified based solely on external appearance. Respondents were also asked about their information-seeking behavior, including whether they had searched for scoliosis-related information on the internet and whether they had visited websites of professional organizations such as the Japanese Scoliosis Society or the Japanese Orthopaedic Association. To evaluate health-seeking behavior, parents were asked whether secondary screening had been recommended after school screening and whether they had attended or planned to attend secondary screening. In addition, respondents were asked whether they would access scoliosis-related information if a QR code were included in the screening questionnaire or in the screening results notice. The questionnaire was developed by the authors based on previous literature and clinical experience with the school scoliosis screening program, and the items were reviewed for content and clarity by orthopedic specialists involved in the program before distribution. The questionnaire was not a previously validated instrument and was not formally pilot-tested before implementation. The full questionnaire is provided in the Appendices.

Statistical analysis

Categorical variables are presented as numbers and percentages. Associations between categorical variables were evaluated using Fisher’s exact test. Odds ratios (ORs) with 95% confidence intervals (CIs) were calculated as effect sizes for the associations of interest. In the analysis of secondary screening attendance, the outcome was intention to attend, defined as a composite of respondents who reported that they had already attended and those who planned to attend; respondents who reported that they had not attended were categorized as having no intention to attend. Because this composite combines confirmed attendance with planned attendance, it does not represent verified follow-up attendance. Respondents with missing data for a given outcome were excluded from the corresponding analysis (complete-case analysis); the denominator for each analysis is reported in the corresponding table. No technical measure was implemented to prevent duplicate submissions of the online questionnaire. A p-value <0.05 was considered statistically significant. All analyses were performed using JMP version 19 (SAS Institute Inc., Cary, NC, USA).

## Results

**Figure 1:**
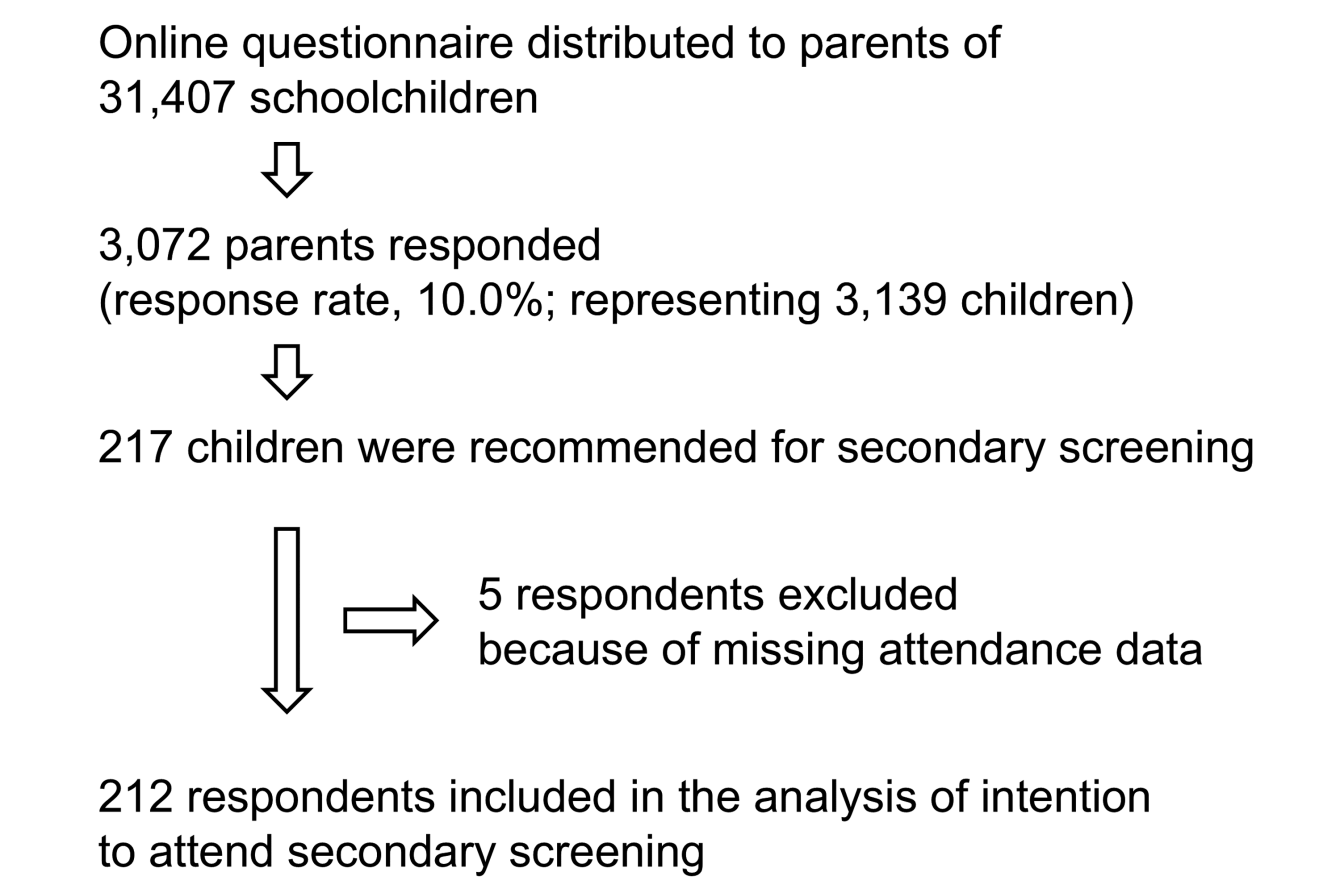
Flow diagram of survey respondents and analysis population. Flow diagram showing the distribution of survey respondents and the analysis population. The online questionnaire was distributed to the parents of 31,407 schoolchildren, and 3,072 parents responded, representing 3,139 children. Among 217 children recommended for secondary screening, 212 respondents with available attendance data were included in the analysis of intention to attend secondary screening.

The flow of respondents and the analysis population is shown in Figure 1.

A total of 3,072 parents responded to the survey, representing 3,139 children, corresponding to a response rate of 10.0%. Among the reported children, 1,523 (48.5%) were boys, and 1,616 (51.5%) were girls. The ages of the children ranged from 5 to 16 years (mean ± standard deviation = 10.3 ± 2.5 years). Among the 3,072 respondents, 1,963 (63.9%) reported that they had been aware of scoliosis before school screening. However, only 887 (28.9%) respondents were aware that scoliosis cannot always be reliably identified based solely on external appearance (Table 1). A total of 648 (21.1%) respondents reported that they had searched for scoliosis-related information on the internet. Among all respondents, 163 (5.3%) had visited the website of the Japanese Scoliosis Society, and 209 (6.8%) had visited the website of the Japanese Orthopaedic Association (Table 1). In the school screening questionnaire, 900 (29.3%) children were reported to have had at least one scoliosis-related item checked. In addition, 217 (7.1%) children were recommended to undergo secondary screening (Table 1).

**Table 1:** Awareness of scoliosis and screening-related findings (n = 3,072).

Variable	Yes, n (%)
Aware of scoliosis before school screening	1,963 (63.9)
Aware that scoliosis cannot be reliably identified based on appearance alone	887 (28.9)
Searched for scoliosis information on the internet	648 (21.1)
Visited the website of the Japanese Scoliosis Society	163 (5.3)
Visited the website of the Japanese Orthopaedic Association	209 (6.8)
Checked at least one scoliosis-related item in the screening questionnaire	900 (29.3)
Recommended for secondary screening	217 (7.1)

Among all respondents, 154 (5.0%) children had attended secondary screening and 69 (2.2%) were planning to attend. In contrast, 413 (13.4%) children had not attended secondary screening, while 2,436 (79.3%) provided no response regarding secondary screening attendance (Table 2). Among the 217 respondents who had been recommended to undergo secondary screening, 146 (67.3%) had attended secondary screening and 52 (24.0%) planned to attend, whereas 14 (6.5%) had not attended (Table 3). Among respondents who had not been recommended for secondary screening (n = 2,855), 8 (0.3%) reported that they had attended secondary screening and 17 (0.6%) planned to attend (Table 4).

**Table 2:** Secondary screening attendance after school screening (n = 3,072).

Variable	n (%)
Attended secondary screening	154 (5.0)
Planned to attend secondary screening	69 (2.2)
Did not attend secondary screening	413 (13.4)
No response	2,436 (79.3)

**Table 3:** Subgroup: respondents recommended for secondary screening (n = 217).

Variable	n (%)
Attended secondary screening	146 (67.3)
Planned to attend secondary screening	52 (24.0)
Did not attend secondary screening	14 (6.5)
No response	5 (2.3)

**Table 4:** Subgroup: respondents not recommended for secondary screening (n = 2,855).

Variable	n (%)
Attended secondary screening	8 (0.3)
Planned to attend secondary screening	17 (0.6)
Did not attend secondary screening	399 (14.0)
No response	2,431 (85.1)

Among the 217 respondents who had been recommended to undergo secondary screening, five (2.3%) respondents did not provide information regarding attendance or planned attendance. Therefore, these five respondents were excluded from the analysis of intention to attend secondary screening, leaving 212 (97.7%) respondents for the analysis shown in Table 5. Among these respondents, internet searching for scoliosis information was significantly associated with intention to attend secondary screening (OR = 5.48, 95% CI = 1.66-18.17; p = 0.006) (Table 5). In contrast, awareness of scoliosis before school screening, awareness that scoliosis cannot be reliably identified based on external appearance alone, checking scoliosis-related items in the screening questionnaire, and visiting websites of professional organizations were not significantly associated with intention to attend secondary screening (Table 5).

**Table 5:** Association between parental awareness and intention to attend secondary screening among respondents recommended for secondary screening who provided attendance data (n = 212). Associations between categorical variables were evaluated using Fisher’s exact test, and ORs with 95% CIs were calculated as the effect size. *: p < 0.05. OR = odds ratio; CI = confidence interval

Variable	Intention to attend, n (%)	No intention, n (%)	OR (95% CI)	P-value
Aware of scoliosis before school screening	126 (59.4)	9 (4.3)	0.97 (0.31–3.01)	1.000
Not aware	72 (34.0)	5 (2.4)	Reference	
Aware that scoliosis cannot be reliably identified by appearance alone	77 (36.3)	121 (57.1)	3.82 (0.83–17.53)	0.087
Not aware	2 (0.9)	12 (5.7)	Reference	
Checked at least one scoliosis-related item in the screening questionnaire	158 (74.5)	10 (4.7)	1.58 (0.47–5.30)	0.496
Did not check	40 (18.9)	4 (1.9)	Reference	
Searched for scoliosis information on the internet	136 (64.2)	4 (1.9)	5.48 (1.66–18.17)	0.006*
Did not search	62 (29.3)	10 (4.7)	Reference	
Visited the website of the Japanese Scoliosis Society	48 (22.6)	1 (0.5)	4.16 (0.53–32.63)	0.197
Did not visit	150 (70.8)	13 (6.1)	Reference	
Visited the website of the Japanese Orthopaedic Association	35 (16.5)	2 (0.9)	1.29 (0.28–6.01)	1.000
Did not visit	163 (76.9)	12 (5.7)	Reference	

Among respondents who had not been recommended for secondary screening but provided responses regarding intention to attend (n = 424), several factors were associated with intention to attend secondary screening (Table 6). Awareness that scoliosis cannot be reliably identified based on external appearance alone (p = 0.021), checking scoliosis-related items in the screening questionnaire (p = 0.028), searching for scoliosis information on the internet (p < 0.001), and visiting the website of the Japanese Scoliosis Society (p < 0.001) were significantly associated with intention to attend secondary screening.

**Table 6:** Association between parental awareness and intention to attend secondary screening among respondents not recommended for secondary screening (n = 424). Associations between categorical variables were evaluated using Fisher’s exact test, and ORs with 95% CIs were calculated as the effect size. *: p < 0.05. OR = odds ratio; CI = confidence interval

Variable	Intention to attend, n (%)	No intention, n (%)	OR (95% CI)	P-value
Aware of scoliosis before school screening	19 (4.5)	251 (59.2)	1.87 (0.73–4.78)	0.207
Not aware	6 (1.4)	148 (34.9)	Reference	
Aware that scoliosis cannot be reliably identified by appearance alone	12 (2.8)	103 (24.3)	2.65 (1.17–6.00)	0.021*
Not aware	13 (3.1)	296 (69.8)	Reference	
Checked at least one scoliosis-related item in the screening questionnaire	11 (2.6)	92 (21.7)	2.62 (1.15–5.97)	0.028*
Did not check	14 (3.3)	307 (72.4)	Reference	
Searched for scoliosis information on the internet	13 (3.1)	69 (16.3)	5.18 (2.27–11.84)	<0.001*
Did not search	12 (2.8)	330 (77.8)	Reference	
Visited the website of the Japanese Scoliosis Society	8 (1.9)	17 (4.0)	10.57 (4.01–27.91)	<0.001*
Did not visit	17 (4.0)	382 (90.1)	Reference	
Visited the website of the Japanese Orthopaedic Association	4 (0.9)	31 (7.3)	2.26 (0.73–7.00)	0.141
Did not visit	21 (5.0)	368 (86.8)	Reference	

When asked whether they would access scoliosis-related information if a QR code were included in the screening questionnaire, 1,654 (53.8%) respondents answered that they would access the website. If a QR code were included in the screening results notice, 2,341 (76.2%) respondents reported that they would access the website (Table 7).

**Table 7:** Willingness to access scoliosis information via QR codes (n = 3,072).

Variable	Yes, n (%)
QR code in the screening questionnaire	1,654 (53.8)
QR code in the screening results notice	2,341 (76.2)

## Discussion

In this questionnaire survey of parents whose children underwent school musculoskeletal screening, several important findings were identified. First, although awareness of scoliosis itself was relatively high, understanding of its clinical characteristics remained limited. Second, internet-based information-seeking behavior was significantly associated with intention to attend secondary screening among respondents who had been recommended for further evaluation. Third, many parents expressed willingness to access scoliosis-related information through QR codes, suggesting that digital information tools may represent a practical approach to supporting parental awareness and appropriate follow-up behavior after school screening.

Although approximately two-thirds of respondents reported that they were aware of scoliosis before school screening, fewer than one-third were aware that scoliosis cannot always be reliably identified based solely on external appearance. Because scoliosis often progresses without pain and early deformities may be subtle, insufficient understanding of this characteristic may lead parents to underestimate the importance of further evaluation [5]. This pattern, relatively high general awareness (63.9%) but limited understanding that scoliosis cannot be reliably identified by appearance alone (28.9%), is consistent with previous reports that public understanding of the clinical features of scoliosis remains limited in many populations [8,9]. Improving parental understanding of the clinical characteristics and potential progression of scoliosis may therefore be important for promoting appropriate healthcare-seeking behavior following school screening.

In the present study, most respondents who had been recommended to undergo secondary screening reported that they had attended or planned to attend secondary screening. This finding suggests that school screening programs may play an important role in promoting appropriate follow-up evaluation for suspected scoliosis. Previous studies have suggested that school scoliosis screening contributes to earlier detection and timely management of scoliosis [12]. A meta-analysis by Fong et al. also demonstrated that school screening facilitates earlier identification of scoliosis and increases the likelihood of nonoperative management such as bracing [13]. Early detection during the growth period is particularly important because timely treatment may prevent curve progression and reduce the need for surgical intervention [3]. However, a small proportion of respondents reported that they had not attended secondary screening despite receiving a recommendation. Although the proportion was relatively small, this finding suggests that factors other than medical recommendation may influence parental decision-making regarding follow-up evaluation. Possible explanations include a perception that the abnormality detected during screening was not serious, the absence of symptoms such as pain, or practical barriers such as limited time to visit a medical institution. Because scoliosis frequently progresses without noticeable symptoms, parents may underestimate the importance of further evaluation if they do not clearly understand the clinical significance of screening findings.

Interestingly, internet-based information-seeking behavior was significantly associated with intention to attend secondary screening among respondents who had been recommended for further evaluation. Parents who had searched for scoliosis-related information on the internet were more likely to report attendance or planned attendance at secondary screening. The observed association between internet information-seeking and intention to attend secondary screening is consistent with previous studies reporting that parents of children with scoliosis frequently use internet resources when seeking health-related information [14] and that parents commonly rely on online health information when making healthcare decisions for their children [11,15]. In addition, adolescents with idiopathic scoliosis and their families often report substantial unmet information and support needs regarding the disease and its management [16]. Similar findings have been reported in other pediatric health contexts, where parental health literacy and access to online medical information influence healthcare utilization and medical decision-making [17]. Improving access to reliable scoliosis-related information may therefore support parents in understanding the importance of follow-up evaluation after school screening.

Another important finding of the present study was the high level of parental interest in accessing scoliosis-related information through QR codes. More than half of the respondents indicated that they would access scoliosis-related information if a QR code were included in the screening questionnaire, and more than three-quarters reported that they would access such information if a QR code were included in the screening results notice. These findings suggest that providing easily accessible digital information may represent a feasible approach to supporting parental understanding of scoliosis following school screening. Digital health communication tools have increasingly been used to deliver medical information efficiently and improve patient engagement [18]. Moreover, improving eHealth literacy has been shown to support informed decision-making and enhance participation in healthcare processes [19]. It should be noted, however, that eHealth literacy and access to online information are not uniform across the population. Parental age, educational level, and socioeconomic status may affect both the ability to locate reliable online information and how that information shapes decisions about follow-up evaluation, so digital educational tools may benefit some parental groups more than others. Because we did not collect parental sociodemographic data, we could not examine these differences, and future studies should assess how digital health literacy affects parental decision-making across sociodemographic groups. Providing QR codes that link to reliable educational resources from professional societies or medical institutions may therefore help parents better understand the clinical significance of screening findings and encourage appropriate healthcare-seeking behavior. Integrating such digital information tools into school screening programs may be a practical way to provide accessible scoliosis-related information to parents. However, because the present study assessed parental willingness to access information rather than the effect of a QR-code-based intervention, prospective studies are needed to determine whether such tools improve actual follow-up attendance after school screening.

First, the response rate was low (10.0%), which represents an important limitation of this study. Parents who were more interested in scoliosis or child health were probably more likely to participate, so respondents likely had higher baseline health awareness and stronger scoliosis-related interest than the general screened population. This selection bias likely inflates the observed levels of parental awareness, information-seeking behavior, and follow-up attendance, and may also have affected the strength of the observed associations. These estimates should therefore be interpreted as hypothesis-generating in this pilot study rather than as accurate population-level prevalences. In addition, parental socioeconomic and educational characteristics were not collected, limiting our ability to assess their influence on the findings. Second, the study relied on self-reported questionnaire data and may therefore be subject to recall bias and reporting bias. In addition, the questionnaire used in this study was not a formally validated instrument and was not pilot-tested before implementation. Although the items were developed based on previous literature and reviewed by orthopedic specialists, the absence of formal validation and pilot testing may have affected the reliability and interpretation of some responses. Future studies using validated and pilot-tested instruments are warranted. Third, a substantial proportion of respondents did not provide answers regarding secondary screening attendance. Because the analyses were conducted after excluding respondents with missing data for this outcome, the results may be subject to non-response bias. Parents who responded to this question may have been more concerned about scoliosis or more likely to seek medical evaluation, which could have influenced the observed associations. Fourth, the cross-sectional design of the study does not allow causal relationships between parental awareness, information-seeking behavior, and secondary screening attendance to be established. In addition, multivariable analysis was not performed; in the subgroup recommended for secondary screening, the number of respondents with no intention to attend was small (14 of 212), which precluded reliable multivariable adjustment, so the reported associations are unadjusted and may be subject to confounding. The questionnaire was also not a formally validated instrument and was not pilot-tested, which may have affected the reliability of some responses. Finally, this survey was conducted in a single city in Japan, and the findings may not necessarily be generalizable to other regions or countries with different school screening systems. Future studies involving larger populations and multiple regions may help clarify the generalizability of these findings and further explore strategies to improve follow-up attendance after school scoliosis screening.

## Conclusions

Detailed understanding of the clinical characteristics of scoliosis remained limited among parents, despite relatively high awareness of the condition. Internet-based information-seeking behavior was associated with intention to attend secondary screening after school screening, suggesting that access to reliable information may play a role in parental decision-making. In addition, many parents expressed willingness to access scoliosis-related information through QR codes, indicating that digital information tools may support parental engagement and appropriate follow-up after school screening. Enhancing access to reliable educational resources may therefore be a feasible way to support parental decision-making, although its effect on actual follow-up attendance should be confirmed in future prospective studies.

## Appendices

Parental awareness of scoliosis and information-seeking behavior following school musculoskeletal screening

Information about your children

Please list the age and sex of all your children who participated in the school musculoskeletal screening.

Child 1

Age: ___ years

Sex:

□ Male

□ Female

Child 2

Age: ___ years

Sex:

□ Male

□ Female

Child 3

Age: ___ years

Sex:

□ Male

□ Female

Child 4

Age: ___ years

Sex:

□ Male

□ Female

Q1. Were you aware of scoliosis before the school musculoskeletal screening?

□ Yes

□ No

Q2. Did you know that scoliosis cannot be reliably diagnosed based only on appearance and may already be advanced when visible deformity becomes obvious?

□ Yes

□ No

Q3. In the musculoskeletal screening questionnaire, did you check any of the scoliosis-related items?

The scoliosis-related items included the following:

Difference in shoulder height

Asymmetry of the waistline

Difference in the height or position of the shoulder blades

Difference in the height of the back when bending forward

□ Yes

□ No

Q4. Were you advised to undergo secondary screening (i.e., to visit an orthopaedic clinic or hospital for further evaluation) after the school musculoskeletal screening?

□ Yes

□ No

Q5. Did your child undergo secondary screening?

□ Yes

□ Planning to undergo

□ No

Q6. If your child did not undergo secondary screening, please indicate the reason. (Multiple answers allowed)

□ Secondary screening was not recommended

□ It did not seem serious

□ The child had no pain

□ No time to visit a hospital

□ Other (please specify):

Q7. Have you ever searched for information about scoliosis on the internet?

□ Yes

□ No

Q8. Which of the following websites have you visited? (Multiple answers allowed)

□ Japanese Scoliosis Society (https://www.sokuwan.jp/patient/)

□ Japanese Orthopaedic Association (https://www.joa.or.jp/public/sick/condition/scoliosis.html)

□ None of the above

Q9. If a QR code linking to a scoliosis information website had been included in the screening questionnaire, would you have accessed it?

□ Yes

□ No

Q10. If a QR code linking to a scoliosis information website had been included in the screening results notice, would you have accessed it?

□ Yes

□ No

Q11. What kind of information would you like to see on the website (e.g., diagnosis, treatment, or the course of scoliosis)? (Multiple answers allowed)
